# Consumption of Dairy Products and Death From Cardiovascular Disease in the Japanese General Population: The NIPPON DATA80

**DOI:** 10.2188/jea.JE20120054

**Published:** 2013-01-05

**Authors:** Imako Kondo, Toshiyuki Ojima, Mieko Nakamura, Shinya Hayasaka, Atsushi Hozawa, Shigeyuki Saitoh, Hirofumi Ohnishi, Hiroshi Akasaka, Takehito Hayakawa, Yoshitaka Murakami, Nagako Okuda, Katsuyuki Miura, Akira Okayama, Hirotsugu Ueshima

**Affiliations:** 1 Faculty of Health Promotional Sciences, Hamamatsu University, Hamamatsu, Shizuoka, Japan; 1 浜松大学健康プロデュース学部; 2 Department of Community Health and Preventive Medicine, Hamamatsu University School of Medicine, Hamamatsu, Shizuoka, Japan; 2 浜松医科大学健康社会医学講座; 3 Department of Preventive Medicine and Epidemiology, Tohoku Medical Megabank Organization, Sendai, Japan; 3 東北メディカルメガバンク機構予防医学・疫学部門; 4 Second Department of Internal Medicine, Sapporo Medical University School of Medicine, Sapporo, Japan; 4 札幌医科大学内科学第二講座; 5 Department of Hygiene and Preventive Medicine, Fukushima Medical University, Fukushima, Japan; 5 福島県立医科大学衛生学・予防医学講座; 6 Department of Medical Statistics, Shiga University of Medical Science, Otsu, Japan; 6 滋賀医科大学医療統計学部門; 7 Department of Nutrition Epidemiology, National Institute of Health and Nutrition, Tokyo, Japan; 7 国立健康・栄養研究所栄養疫学研究部; 8 Department of Health Science, Shiga University of Medical Science, Otsu, Japan; 8 滋賀医科大学公衆衛生学部門; 9 The First Institute for Health Promotion and Health Care, Japan Anti-Tuberculosis Association, Tokyo, Japan; 9 日本結核予防会第一健康相談所; 10 Lifestyle-Related Disease Prevention Center, Shiga University of Medical Science, Otsu, Japan; 10 滋賀医科大学生活習慣病予防センター

**Keywords:** dairy products, cardiovascular disease, mortality, blood pressure, coronary heart disease

## Abstract

**Background:**

Recent Western studies show an inverse association between milk and dairy product intake and cardiovascular disease (CVD). We studied the association between consumption of milk and dairy products and CVD death in Japan.

**Methods:**

Men and women aged 30 years or older were followed for 24 years. All had participated in a national nutrition survey in 300 health districts throughout Japan in 1980. The Cox proportional hazards model was used to assess mortality risk according to tertiles of milk and dairy product intake, with the high consumption group as reference. Hazard ratios (HRs) per 100-g/day increase in consumption were also estimated.

**Results:**

During the 24-year follow-up period, there were 893 CVD deaths, 174 deaths from coronary heart disease (CHD), and 417 stroke deaths among 9243 participants. For women, the HRs for death from CVD, CHD, and stroke in the low consumption group were 1.27 (95% CI: 0.99–1.58; *P* for trend = 0.045), 1.67 (0.99–2.80; *P* = 0.02), and 1.34 (0.94–1.90; *P* = 0.08), respectively, after adjustment for age, body mass index, smoking status, alcohol drinking habits, history of diabetes, use of antihypertensives, work category, and total energy intake. With each 100-g/day increase in consumption of milk and dairy products, HRs tended to decrease for deaths from CVD (HR, 0.86; 95% CI, 0.74–0.99), CHD (0.73; 0.52–1.03), and stroke (0.81; 0.65–1.01) in women. No significant association was observed in men.

**Conclusions:**

Consumption of milk and dairy products was inversely associated with CVD death among women in Japan.

## Abstract (ja)

**【背景】:**

近年、牛乳・乳製品の摂取と循環器疾患との負の関連が西洋諸国の研究で報告されている。本研究では、日本における牛乳・乳製品の摂取と循環器疾患死亡との関連について検討した。

**【方法】:**

1980年に日本全体の300地区で実施した国民栄養調査の対象者のうち30歳以上の男女を24年間追跡した。牛乳・乳製品摂取量の3分位間の死亡リスクをコックスの比例ハザードモデルにより高摂取群を基準として算出した。また、摂取量1日100ｇ増加あたりのハザード比も算出した。

**【結果】:**

対象者9,243人の24年間の追跡期間における循環器疾患死亡は893人で、そのうち心疾患死亡が174人、脳血管疾患死亡が417人であった。女性において、低摂取群の循環器疾患死亡、心疾患死亡、脳血管疾患死亡のハザード比は年齢、BMI、喫煙習慣、飲酒習慣、糖尿病歴、降圧剤服薬、職業区分および総エネルギー摂取量を調整した場合それぞれ1.27(95%信頼区間:0.99-1.58;トレンドp=0.045)、1.67(0.99-2.80;p=0.02)、1.34(0.94-1.90;p=0.08)であった。牛乳・乳製品1日100ｇ摂取増加毎のハザード比は、女性において循環器疾患死亡0.86(0.74-0.99)、心疾患死亡 0.73(0.52-1.03)、脳血管疾患死亡 0.81(0.65-1.01)で低下傾向にあった。男性では有意な関連は見られなかった。

**【結論】:**

牛乳・乳製品の摂取は日本において女性で循環器疾患死亡と負の関連があった。

## INTRODUCTION

In recent years, findings from several cohort studies have suggested that intake of dairy products is inversely associated with the risk of hypertension,^1^^,^^2^ cardiovascular disease (CVD),^3^ coronary heart disease (CHD),^4^^,^^5^ and stroke.^5^^–^^7^ In addition, a meta-analysis^8^ of 4 cohort studies from Western countries indicated that milk intake may be inversely associated with overall CVD risk.

In Japan, consumption of milk and dairy products is recommended to ensure nutritional balance, especially adequate intake of calcium. New dietary guidelines in Japan^9^ encourage individuals to obtain sufficient calcium by consuming milk and dairy products, green and yellow vegetables, tofu, and small fish. Similarly, the Japanese Food Guide Spinning Top,^10^ a dietary balance guideline for Japanese, recommends consumption of 2 servings of milk and dairy products (200 ml of milk, equivalent to 200 mg of calcium) per day for adults. However, current consumption of milk and dairy products in Japan is much lower than this recommended level. The National Nutrition Survey, Japan 2008 (NNSJ 2008)^11^ found that the average daily consumption of milk and dairy products by adults was 89.0 g, which is much lower than in Western countries.

Most of the above-mentioned studies that showed an inverse association with CVD were conducted in Western countries, where milk and dairy product consumption is much higher than in Japan. Only 2 cohort studies^6^^,^^7^ of the association between dairy calcium intake and CVD risk have been done in Japan. In these 2 studies, participants were followed for about 10 to 13 years.

We analyzed the association of milk and dairy product consumption with CVD death in a representative sample of Japanese who were followed for 24 years.

## METHODS

### Dataset

For data analysis, we used an integrated dataset from the NIPPON DATA80 (National Integrated Project for Prospective Observation of Non-communicable Disease And its Trends in the Aged). The participants in this cohort were the same as those in the 1980 National Survey on Circulatory Disorders.^12^ A total of 10 546 community-based men and women aged 30 years or older in 300 randomly selected districts throughout Japan participated in the survey, which consisted of history-taking, physical examinations, blood tests, and self-administered questionnaires on lifestyle. Details of NIPPON DATA80 have been described previously.^13^^–^^15^

The NIPPON DATA80 cohort also participated in the National Nutrition Survey, Japan, conducted in the same year (NNSJ1980),^16^ and data on nutritional intake per individual were added to the dataset from the results of NNSJ1980. Nutrient intake was estimated using weighed diet records for 3 consecutive days during which there were no special events. Weekends and holidays were avoided for the survey. Registered dietitians at public health centers in each district, who were specially trained in dietary interviews, visited participants’ homes at least once during the survey. Diet records were thoroughly reviewed by the registered dietitians. Nutrient intake was calculated using the updated Standard Tables for Food Composition in Japan, Fourth Edition, with matched fatty acid values and micronutrients. Food and nutrient intakes of each household member were estimated by dividing the household intake data from NNSJ1980,^16^ using average intake by sex and age group calculated from NNSJ1995.^17^ The average intake of NNSJ1995^17^ was calculated using a method that combined household-based weighed diet records with an approximation of proportions by which family members shared each dish or food in the household. For each participant, means of the estimated individual food and nutrient intakes for 3 consecutive days were used in the analyses. The details of the methods used in the nutritional survey and the estimation of individual intake have been described elsewhere.^18^

### Baseline variables

Height in stocking feet and weight in light clothing were measured. Body mass index (BMI) was calculated as weight (kg) divided by the square of the height (m). Blood pressure was measured on the right arm of seated participants by trained observers using a standard mercury sphygmomanometer. Serum total cholesterol was analyzed using an autoanalyzer (SMA12/60; Technicon, Tarrytown, NY, USA) at 1 central laboratory (present name: Osaka Medical Center for Health Science and Promotion). Since April 1975, the precision and accuracy of the cholesterol measurements in the laboratory have been certified by the Centers for Disease Control–National Heart, Lung, and Blood Institute (CDC-NHLBI) Lipid Standardization Program of the Centers for Disease Control and Prevention (CDC) in Atlanta, Georgia.

Information on history of CVD and/or diabetes, use of antihypertensives, smoking status, drinking habits, and work category was obtained from interviewer-administered questionnaires. Smoking status was classified as never-smoker, current smoker of 20 or fewer cigarettes/day, current smoker of 21 or more cigarettes/day, and ex-smoker. Alcohol drinking habit was classified as never-drinker, occasional drinker, daily drinker, and ex-drinker. Work category was classified as manager/professional and non-manager/professional. Milk and dairy product consumption per day was used to allocate participants into 3 groups by sex-specific tertiles. In men, daily consumption of 42.4 g or less was considered low intake, 42.5 to 81.5 as moderate intake, and 81.6 g or more as high intake. In women, the respective levels were 53.6 g or less, 53.7 to 105.6 g, and 105.7 g or more. In the present study, data on milk and dairy products cannot be divided into type of milk and dairy products. In 1980, almost all (93%) milk and dairy product consumption was in the form of milk.^16^

### Outcome

Outcome data obtained from follow-up surveys until 2004 were analyzed in this study. Underlying causes of death were coded for Japanese National Vital Statistics according to the Ninth International Classification of Disease (ICD-9) until the end of 1994 and according to the 10th International Classification of Disease (ICD-10) from the beginning of 1995. Permission to use National Vital Statistics was obtained from the Management and Coordination Agency of the Government of Japan. The respective codes for ICD-9 and ICD-10 were as follows: CVD—393 to 459 (ICD-9) and I00 to I99 (ICD-10); CHD—410 to 414 (ICD-9) and I20 to I25 (ICD-10); stroke—430 to 438 (ICD-9) and I60 to I69 (ICD-10). Approval for this study was obtained from the Institutional Review Board of Shiga University of Medical Science.

### Statistical analyses

We excluded participants with a total energy intake less than 500 kcal/day or greater than 5000 kcal/day (*n* = 139), those lost to follow-up (*n* = 909), those with a past history of CVD (*n* = 280), and those with missing data on height, weight, blood pressure, serum total cholesterol, history of diabetes, smoking status, or alcohol drinking habit (*n* = 48).

First, variables were compared between tertiles of milk and dairy product consumption. One-way analysis of variance was used to compare means of milk and dairy product consumption, age, BMI, systolic blood pressure, diastolic blood pressure, serum total cholesterol concentration, total energy intake, and consumption of foods (vegetables, fruit, fish and shellfish, meat and poultry, eggs, fat and oil, and salt) among tertiles by sex. The chi-square test was used to compare the categorical variables of history of diabetes, use of antihypertensives, smoking status, alcohol drinking habit, and work category among the tertiles by sex. Analysis of covariance was used to compare age-adjusted means for milk and dairy product consumption, BMI, systolic blood pressure, diastolic blood pressure, serum total cholesterol concentration, total energy intake, and consumption of foods among tertiles by sex.

Second, crude mortality of CVD, CHD, and stroke among tertiles was calculated, and the Cox proportional hazards model was then used to estimate hazard ratios (HRs) of CVD, CHD, and stroke mortality associated with milk and dairy product consumption by sex. HRs and their 95% CIs for the moderate and low milk and dairy product intake groups were calculated using the high intake group as a reference. The trend tests were performed using the Cox proportional hazards model, with the milk and dairy consumption tertiles analyzed as a continuous variable. Also, HRs per 100-g/day increase in milk and dairy product consumption were estimated in both sexes.

We constructed 3 models: age was adjusted in Model 1; BMI (sex-specific tertiles), alcohol drinking habit (never, occasional, daily, and ex-drinker), smoking status (never, current smoker of ≤20 and ≥21 cigarettes/day, and ex-smoker), history of diabetes (yes, no), use of antihypertensives (yes, no), work category (manager/professional, non-manager/professional) as dummy variables, and total energy intake as a continuous variable, were further adjusted in Model 2; and systolic blood pressure (≤119, 120–139, 140–159, and ≥160 mm Hg) and serum total cholesterol (≤129, 130–199, 200–219, and ≥220 mg/dl) as dummy variables, and consumption of vegetable, fruit, fish and shellfish, meat and poultry, eggs, fat and oil, and salt as continuous variables, were further adjusted in Model 3. We defined Model 2 as the principal model and Model 3 as sensitivity analysis, because systolic blood pressure, serum total cholesterol, and food intake, which were adjusted in Model 3, may be intermediate factors. Thus, the possibility of overadjustment in Model 3 cannot be denied. All statistical analyses were performed using SPSS for Windows version 18.0J, SPSS, Tokyo, Japan. All values were 2-tailed, and *P* < 0.05 was considered to indicate statistical significance.

## RESULTS

The analyzed participants were 9243 people (4045 men, and 5198 women), and their characteristics at baseline are shown in Table 1 by sex-specific tertile of milk and dairy product intake. There were significant differences among groups in milk and dairy product consumption, age, systolic blood pressure, serum total cholesterol, work category, total energy intake, and consumption of vegetables, fruit, meat and poultry, eggs, fat and oil, and salt (men and women); in history of diabetes and smoking status (men only); and in BMI, diastolic blood pressure, use of antihypertensives, and alcohol drinking habit (women only).

**Table 1.** Baseline characteristics of participants by tertiles of milk and dairy product consumption (NIPPON DATA80, 1980)

		Low	Moderate	High	*P* value	

Characteristic					*P*^a^	*P*^b^
Men						
*n*		1349	1349	1347		
Range (g/d)		0–42.4	42.5–81.5	81.6–630.5		
Milk and dairy product consumption (g/d)		18.0 ± 15.0	61.5 ± 11.1	132.6 ± 53.3	<0.01	<0.01
Age (years)		52.1 ± 13.0	47.9 ± 12.5	50.8 ± 13.5	<0.01	
BMI (kg/m^2^)		22.5 ± 2.8	22.5 ± 2.9	22.6 ± 2.9	0.35	0.09
Systolic blood pressure (mm Hg)		140.6 ± 21.7	136.6 ± 20.3	137.8 ± 20.2	<0.01	0.02
Diastolic blood pressure (mm Hg)		84.2 ± 12.6	83.1 ± 12.3	83.3 ± 12.0	0.06	0.31
Serum total cholesterol (mg/dl)		182.9 ± 32.9	186.2 ± 32.5	189.5 ± 32.8	<0.01	<0.01
History of diabetes (%)		3.9	3.3	5.4	0.02	
Use of antihypertensives (%)		10.1	8.2	10.7	0.07	

Smoking status	Never-smoker (%)	16.3	18.2	20.9	<0.01	
	≤20 cigarettes/day (%)	42.3	36.8	36.7		
	≥21 cigarettes/day (%)	24.3	27.9	22.2		
	Ex-smoker (%)	17.0	17.0	20.2		

Alcohol drinking habits	Never-drinker (%)	19.3	19.8	20.9	0.21	
	Occasional drinker (%)	24.2	27.7	27.5		
	Daily drinker (%)	51.1	47.4	46.3		
	Ex-drinker (%)	5.3	5.2	5.3		

Manager/professional (%)		41.1	50.3	51.4	<0.01	
Total energy intake (kcal/d)		2314 ± 489	2398 ± 460	2505 ± 464	<0.01	<0.01
Vegetable consumption (g/d)		284.8 ± 125.0	278.1 ± 101.7	299.0 ± 112.3	<0.01	<0.01
Fruit consumption (g/d)		121.7 ± 96.9	133.8 ± 86.9	168.7 ± 100.4	<0.01	<0.01
Fish and shellfish consumption (g/d)		124.8 ± 66.5	122.9 ± 63.0	127.5 ± 62.1	0.17	0.18
Meat and poultry consumption (g/d)		64.5 ± 41.3	73.8 ± 43.1	73.4 ± 39.8	<0.01	<0.01
Egg consumption (g/d)		37.1 ± 23.1	40.4 ± 20.6	44.3 ± 22.4	<0.01	<0.01
Fat and oil consumption (g/d)		14.7 ± 11.1	17.4 ± 11.1	18.9 ± 12.5	<0.01	<0.01
Salt consumption (g/d)		15.7 ± 6.2	14.7 ± 5.1	15.1 ± 5.2	<0.01	<0.01

Women						
*n*		1733	1734	1731		
Range (g/d)		0–53.6	53.7–105.6	105.7–577.5		
Milk and dairy product consumption (g/d)		22.7 ± 18.9	79.0 ± 14.8	168.3 ± 62.2	<0.01	<0.01
Age (years)		54.4 ± 13.1	49.0 ± 13.7	48.8 ± 12.4	<0.01	
BMI (kg/m^2^)		23.1 ± 3.4	22.9 ± 3.4	22.6 ± 3.2	<0.01	<0.01
Systolic blood pressure (mm Hg)		138.0 ± 22.6	132.0 ± 20.6	131.6 ± 20.3	<0.01	<0.01
Diastolic blood pressure (mm Hg)		81.2 ± 12.2	78.6 ± 11.6	78.9 ± 11.6	<0.01	<0.01
Serum total cholesterol (mg/dl)		190.8 ± 33.6	187.1 ± 33.8	194.3 ± 34.6	<0.01	<0.01
History of diabetes (%)		1.5	2.2	2.6	0.07	
Use of antihypertensives (%)		14.5	9.4	10.0	<0.01	

Smoking status	Never-smoker (%)	87.9	89.1	90.2	0.37	
	≤20 cigarettes/day (%)	9.1	8.1	6.9		
	≥21 cigarettes/day (%)	0.6	0.7	0.8		
	Ex-smoker (%)	2.4	2.0	2.1		

Alcohol drinking habits	Never-drinker (%)	80.2	78.3	77.1	0.02	
	Occasional drinker (%)	14.8	18.0	18.7		
	Daily drinker (%)	3.3	2.7	2.5		
	Ex-drinker (%)	1.7	1.0	1.7		

Manager/professional (%)		8.7	13.4	13.9	<0.01	
Total energy intake (kcal/d)		1861 ± 408	1898 ± 362	2036 ± 376	<0.01	<0.01
Vegetable consumption (g/d)		269.1 ± 118.1	256.4 ± 94.8	280.1 ± 107.2	<0.01	<0.01
Fruit consumption (g/d)		165.0 ± 128.7	174.8 ± 111.1	222.1 ± 130.3	<0.01	<0.01
Fish and shellfish consumption (g/d)		98.7 ± 52.1	93.9 ± 44.9	96.3 ± 47.2	0.01	0.08
Meat and poultry consumption (g/d)		48.7 ± 37.5	54.0 ± 32.5	58.3 ± 31.8	<0.01	<0.01
Egg consumption (g/d)		31.5 ± 20.1	34.4 ± 18.1	38.5 ± 20.1	<0.01	<0.01
Fat and oil consumption (g/d)		13.0 ± 9.8	15.1 ± 9.4	17.9 ± 11.2	<0.01	<0.01
Salt consumption (g/d)		13.5 ± 5.3	12.6 ± 4.3	13.0 ± 4.5	<0.01	<0.01

^a^Tested by 1-way analysis of variance for continuous variables and by the chi-square test for categorical variables.

^b^Tested by analysis of covariance for continuous variables; adjusted for age.

Abbreviation: BMI, body mass index.

During the 24-year follow-up period, there were 2580 all-cause deaths (1372 men, 1208 women) and 893 CVD deaths (440 men, 453 women), of which 174 were CHD deaths (85 men, 89 women) and 417 were stroke deaths (217 men, 200 women).

The HRs for CVD death by tertile are shown in Table 2. For CVD death, the HR was marginally higher in the low consumption group in Model 2 among women (HR 1.27; 95% CI 0.99–1.58). The HR for CVD death significantly increased with decreasing milk and dairy product consumption in Model 2 among women (*P* for trend = 0.045). There was no significant association of milk and dairy product consumption with CVD death in men. For CHD death, the HR was marginally higher in the low consumption group in Model 2 among women (HR, 1.67; 95% CI, 0.99–2.80). The HRs for CHD death significantly increased with decreasing milk and dairy product consumption in all models (*P* for trend: Model 1, *P* = 0.03; Model 2; *P* = 0.02; Model 3, *P* = 0.045; data from Model 3 not shown). In men, there was no significant association of milk and dairy product consumption with CHD death. Milk and dairy product consumption was not significantly associated with stroke death in men or women.

**Table 2.** Hazard ratios (95% CI) for mortality associated with milk and dairy product consumption (NIPPON DATA80, 1980–2004)

	Low	Moderate	High	*P* for trend
Men				
Person-years	26 861	28 419	27 100	
Cardiovascular disease				
Number of deaths	158	111	171	
Model 1^a^	0.90 (0.72–1.11)	0.89 (0.70–1.13)	1 (reference)	0.32
Model 2^b^	0.89 (0.72–1.11)	0.90 (0.71–1.15)	1 (reference)	0.31
Coronary heart disease				
Number of deaths	28	18	39	
Model 1^a^	0.69 (0.43–1.13)	0.59 (0.34–1.04)	1 (reference)	0.13
Model 2^b^	0.67 (0.41–1.11)	0.57 (0.32–1.01)	1 (reference)	0.11
Stroke				
Number of deaths	84	57	76	
Model 1^a^	1.07 (0.79–1.46)	1.04 (0.73–1.46)	1 (reference)	0.66
Model 2^b^	1.10 (0.80–1.50)	1.09 (0.77–1.54)	1 (reference)	0.58
Women				
Person-years	35 996	37 516	37 904	
Cardiovascular disease				
Number of deaths	215	127	111	
Model 1^a^	1.21 (0.96–1.52)	0.99 (0.77–1.28)	1 (reference)	0.07
Model 2^b^	1.27 (0.99–1.58)	1.03 (0.79–1.33)	1 (reference)	0.05
Coronary heart disease				
Number of deaths	51	17	21	
Model 1^a^	1.56 (0.94–2.60)	0.71 (0.37–1.35)	1 (reference)	0.03
Model 2^b^	1.67 (0.99–2.80)	0.72 (0.38–1.36)	1 (reference)	0.02
Stroke				
Number of deaths	98	54	48	
Model 1^a^	1.28 (0.91–1.82)	0.98 (0.66–1.45)	1 (reference)	0.12
Model 2^b^	1.34 (0.94–1.90)	1.04 (0.70–1.54)	1 (reference)	0.08

^a^Adjusted for age.

^b^Adjusted for age, body mass index, smoking status, alcohol drinking habit, history of diabetes, use of antihypertensives, work category, and total energy intake.

The HRs for death from CVD, CHD, and stroke per 100-g/day increase in consumption of milk and dairy products are shown in Table 3. In Model 2, CVD death in women significantly decreased with each 100-g/day increase in milk and dairy product consumption (HR, 0.86; 95% CI, 0.74–0.99). The results were not materially different in women aged 50 years or older (data not shown).

**Table 3.** Hazard ratios (95% CI) for mortality per 100-g/day increase in milk and dairy product consumption (NIPPON DATA80, 1980–2004)

	Model 1^a^	Model 2^b^
Men		
Cardiovascular disease	1.01 (0.88–1.17)	1.03 (0.89–1.20)
Coronary heart disease	1.24 (0.93–1.66)	1.33 (0.97–1.82)
Stroke	0.93 (0.75–1.14)	0.91 (0.74–1.13)
Women		
Cardiovascular disease	0.87 (0.76–1.01)	0.86 (0.74–0.99)
Coronary heart disease	0.76 (0.54–1.07)	0.73 (0.52–1.03)
Stroke	0.83 (0.67–1.03)	0.81 (0.65–1.01)

^a^Adjusted for age.

^b^Adjusted for age, body mass index, smoking status, alcohol drinking habit, history of diabetes, use of untihypertensives, work category, and total energy intake.

## DISCUSSION

A 24-year follow-up of Japanese participants showed that consumption of milk and dairy products was inversely associated with CVD death in women. No significant associations were observed in men.

An inverse association between dairy product consumption and CVD mortality was previously reported. Ness et al conducted a 25-year follow-up study of men in Scotland and found that milk consumption was inversely associated with death from all causes, CVD, and CHD.^4^ In a cohort study of men, Elwood et al found that milk consumption was associated with lower levels of ischemic heart disease and stroke.^5^ In a study of Australian adults who were followed for an average of 14.4 years, Bonthuis et al noted that full-fat dairy intake was inversely associated with CHD death.^3^ In the Japan Collaborative Cohort study (JACC)^6^ and the Japan Public Health Center study (JPHC),^7^ calcium intake from dairy products was associated with lower stroke mortality in men and women, but no inverse association was seen for CHD death. Among these studies, the results from JACC and JPHC differ somewhat from the present results. In a systematic review of 10 cohort studies, however, Elwood et al suggested that milk drinking is associated with a small but meaningful reduction in vascular disease risk.^19^

In the present study, consumption of milk and dairy products was associated with CVD death only in women; no significant associations were seen in men. However, men consumed far less milk and dairy products than did women in the present study. Furthermore, levels of milk and dairy product consumption in the present study were much lower than those reported in Western studies,^4^^,^^5^ which observed an inverse association in men. For example, in the present study, daily consumption in the highest tertile of men was 81.6 g or more. In contrast, Ness et al reported^4^ a daily consumption of 189 ml or less in the lowest tertile of participants. Had the men in the present study consumed much more milk and dairy products, the association of milk and dairy product consumption with mortality may have changed. Another reason for our findings in men is that men had a greater number of other risk factors, such as work stress, that may be more strongly related to CHD.^20^ In the present study, although work category was adjusted for in the analysis, unidentified confounding factors may have remained.

Serum total cholesterol level was higher in the high intake group than in the lower intake groups. Serum cholesterol is a contributing factor for CVD,^21^ and despite the high serum total cholesterol concentration in the high intake group, consumption of milk and dairy products was associated with lower CVD mortality in women. Model 3 further adjusted for serum total cholesterol, systolic blood pressure, and food intakes, which may be confounding factors or intermediate factors; however, the inverse association of milk and dairy product intake with CHD death was not substantially changed. This study began in 1980, and statin anticholesterol agents began to be marketed in Japan in 1989. Thus, statin use could be masking the effects of serum total cholesterol to some degree. In our previous cross-sectional study,^22^ the regression coefficients between serum total cholesterol levels and milk and dairy products and other food groups were lower in the 1990s than in the 1980s.

We also found that blood pressure was lower in the high intake group than in the lower intake groups. Some studies have shown an inverse association between consumption of dairy products and blood pressure.^1^^,^^2^^,^^23^^,^^24^ In a meta-analysis of randomized controlled trials, Van Mierlo et al suggested that calcium supplementation reduced blood pressure.^25^ In an assessment of 2 cross-sectional studies (INTERMAP and INTERSALT), Kesteloot et al suggested that altered calcium homeostasis, as shown by increased calcium excretion, is associated with higher blood pressures.^26^ In their meta-analysis of randomized controlled trials, Jia-Ying Xu et al showed that milk-derived tripeptides have hypotensive effects in prehypertensive and hypertensive adults.^27^ Reports of an inverse association of milk and its ingredients with blood pressure are increasing; however, the association remains controversial.

It is unclear why there was an inverse association of milk and dairy product consumption with CVD death in the present study. Further research is warranted to elucidate the mechanisms involved. The same view was presented in the above-mentioned study showing an inverse association of CVD with milk and dairy products.^3^ Moreover, although Elwood et al suggested that randomized controlled studies of dietary factors and disease risk would yield the best evidence, adequately powered randomized controlled trials of milk consumption would be impossible.^5^ Two cohort studies^28^^,^^29^ in Japan suggested that intakes of animal products such as eggs, dairy products, and fish were inversely associated with stroke risk. Among the present participants, those who consumed more milk also consumed more vegetables and fruits and moderate amounts of salt. To some extent, high milk and dairy product consumption may be an indicator of a healthy diet among Japanese.

The advantages of the present study were that it investigated a large, randomly selected sample of the general population of Japan, that it was a cohort study with 24 years of follow-up, that it was conducted in a region with low milk and dairy product consumption (in contrast to Western countries), and that milk and dairy product intake was based on weighed records, which is considered the gold standard for determining the weight of consumed foods.

The limitations of our study were that assessment of milk and dairy product intake at baseline may not have been representative of the full 24 years of the follow-up period. In addition, the calculated consumption at baseline may not have been the usual intake, because the food intake survey was based on records for only 3 days, excluding weekends and holidays.

Some studies analyzed food consumption by type of milk and dairy product, such as cheese and yogurt,^3^^,^^24^ or by fat content.^1^^–^^3^^,^^24^ The present analysis was done with milk and dairy products as a single group. However, daily consumption of milk and dairy products per person in 1980 was 115.2 g, with milk accounting for 107.2 g.^16^ In recent years, the variety of milk and dairy products has increased, so future Japanese studies will likely analyze consumption by type of dairy product. Jenum et al reported that cardiovascular risk factors and population-level socioeconomic characteristics were strongly related to mortality.^30^ Thus, future studies may need to analyze social background.

In conclusion, consumption of milk and dairy products (in the present research, almost entirely milk) was inversely associated with CVD death in women but not in men. Each 100-g/day increase in milk and dairy product consumption significantly decreased the HR for CVD death by 14%. No significant associations were observed in men. In Japan, milk and dairy product consumption has mainly been recommended for the purpose of increasing calcium intake but should be further investigated for its nutritional role in the Japanese diet.

## ONLINE ONLY MATERIALS

The Japanese-language abstract for articles can be accessed by clicking on the tab labeled Supplementary materials at the journal website http://dx.doi.org/10.2188/jea.JE20120054.

Abstract in Japanese.

## APPENDIX

### The NIPPON DATA80/90 Research Group

Chairperson: Hirotsugu Ueshima (Department of Health Science, Shiga University of Medical Science, Otsu, Shiga).

Co-chairpersons: Akira Okayama (The First Institute for Health Promotion and Health Care, Japan Anti-Tuberculosis Association, Tokyo) for the NIPPON DATA80, and Tomonori Okamura (Department of Preventive Medicine and Public Health, Keio University, Tokyo) for the NIPPON DATA90.

Research members: Shigeyuki Saitoh (Department of Second Internal Medicine, Sapporo Medical University, Sapporo, Hokkaido), Kiyomi Sakata (Department of Hygiene and Preventive Medicine, Iwate Medical University, Morioka, Iwate), Atsushi Hozawa (Department of Preventive Medicine and Epidemiology, Tohoku Medical Megabank Organization, Tohoku University, Sendai, Miyagi), Takehito Hayakawa (Department of Hygiene and Preventive Medicine, Fukushima Medical University, Fukushima), Yosikazu Nakamura (Department of Public Health, Jichi Medical University, Shimotsuke, Tochigi), Yasuhiro Matsumura (Faculty of Health and Nutrition, Bunkyo University, Chigasaki, Kanagawa), Nobuo Nishi (Project for the National Health and Nutrition Survey, National Institute of Health and Nutrition, Tokyo), Nagako Okuda (Section of Shokuiku, Department of Nutritional Education, National Institute of Health and Nutrition, Tokyo), Fumiyoshi Kasagi (Institute of Radiation Epidemiology, Radiation Effects Association, Tokyo), Toru Izumi (Faculty of Medicine, Kitasato University, Sagamihara, Kanagawa), Toshiyuki Ojima (Department of Community Health and Preventive Medicine, Hamamatsu University School of Medicine, Hamamatsu, Shizuoka), Koji Tamakoshi (Department of Public Health and Health Information Dynamics, Nagoya University Graduate School of Medicine, Nagoya, Aichi), Hideaki Nakagawa (Department of Epidemiology and Public Health, Kanazawa Medical University, Kanazawa, Ishikawa), Katsuyuki Miura, Takayoshi Ohkubo, Yoshikuni Kita, Yasuyuki Nakamura (Cardiovascular Epidemiology, Kyoto Women’s University, Kyoto), Katsushi Yoshita (Osaka City University Graduate School of Human Life Science, Osaka), Aya Kadota (Department of School Nursing and Health Education, Osaka Kyoiku University, Kashiwara, Osaka), Kazunori Kodama (Radiation Effects Research Foundation, Hiroshima) and Yutaka Kiyohara (Department of Environmental Medicine, Kyushu University, Fukuoka).

## References

[1] Wang L, Manson JE, Buring JE, Lee IM, Sesso HD Dietary intake of dairy products, calcium, and vitamin D and the risk of hypertension in middle-aged and older women. Hypertension. 2008;51:1073–9 10.1161/HYPERTENSIONAHA.107.107821 18259007

[2] Alonso A, Beunza JJ, Delgado-Rodríguez M, Martínez JA, Martínez-González MA Low-fat dairy consumption and reduced risk of hypertension: the Seguimiento Universidad de Navarra (SUN) cohort. Am J Clin Nutr. 2005;82:972–9 16280427 10.1093/ajcn/82.5.972

[3] Bonthuis M, Hughes MC, Ibiebele TI, Green AC, van der Pols JC Dairy consumption and patterns of mortality of Australian adults. Eur J Clin Nutr. 2010;64:569–77 10.1038/ejcn.2010.45 20372173

[4] Ness AR, Smith GD, Hart C Milk, coronary heart disease and mortality. J Epidemiol Community Health. 2001;55:379–82 10.1136/jech.55.6.379 11350992 PMC1731907

[5] Elwood PC, Strain JJ, Robson PJ, Fehily AM, Hughes J, Pickering J, Milk consumption, stroke, and heart attack risk: evidence from the Caerphilly cohort of older men. J Epidemiol Community Health. 2005;59:502–5 10.1136/jech.2004.027904 15911647 PMC1757052

[6] Umesawa M, Iso H, Date C, Yamamoto A, Toyoshima H, Watanabe Y, Dietary intake of calcium in relation to mortality from cardiovascular disease: the JACC Study. Stroke. 2006;37:20–6 10.1161/01.STR.0000195155.21143.38 16339476

[7] Umesawa M, Iso H, Ishihara J, Saito I, Kokubo Y, Inoue M, Dietary calcium intake and risks of stroke, its subtypes, and coronary heart disease in Japanese: the JPHC Study Cohort I. Stroke. 2008;39:2449–56 10.1161/STROKEAHA.107.512236 18635855

[8] Soedamah-Muthu SS, Ding EL, Al-Delaimy WK, Hu FB, Engberink MF, Willett WC, Milk and dairy consumption and incidence of cardiovascular diseases and all-cause mortality: dose-response meta-analysis of prospective cohort studies. Am J Clin Nutr. 2011;93:158–71 10.3945/ajcn.2010.29866 21068345 PMC3361013

[9] Ministry of Education, Culture, Sports, Science and Technology; Ministry of Health and Welfare; Ministry of Agriculture, Forestry and Fisheries, Japan. Dietary guidelines for health promotion in Japan 2000. Tokyo: Daiichi Shuppan; 2003 (in Japanese).

[10] Ministry of Health and Welfare; Ministry of Agriculture, Forestry and Fisheries, Japan. Japanese Food Guide Spinning Top. Tokyo: Daiichi Shuppan; 2006 (in Japanese).

[11] Ministry of Health and Welfare, Japan. The National Health and Nutrition Survey in Japan, 2008. Tokyo: Daiichi Shuppan; 2011 (in Japanese).

[12] Ministry of Health and Welfare, Japan. The National Survey on Circulatory Disorders, 1980. Tokyo: Japan Heart Foundation; 1982 (in Japanese).

[13] Okamura T, Kadowaki T, Hayakawa T, Kita Y, Okayama A, Ueshima H; NIPPON DATA80 Research Group What cause of mortality can we predict by cholesterol screening in the Japanese general population? J Intern Med. 2003;253:169–80 10.1046/j.1365-2796.2003.01080.x 12542557

[14] NIPPON DATA80 Research Group Risk assessment chart for death from cardiovascular disease based on a 19-year follow-up study of a Japanese representative population. Circ J. 2006;70:1249–55 10.1253/circj.70.1249 16998254

[15] Ueshima H, Choudhury SR, Okayama A, Hayakawa T, Kita Y, Kadowaki T, Cigarette smoking as a risk factor for stroke death in Japan: NIPPON DATA80. Stroke. 2004;35:1836–41 10.1161/01.STR.0000131747.84423.74 15166389

[16] Ministry of Health and Welfare, Japan. The National Nutrition Survey in Japan, 1980. Tokyo: Daiichi Shuppan; 1982 (in Japanese).

[17] Ministry of Health and Welfare, Japan. The National Nutrition Survey in Japan, 1995. Tokyo: Daiichi Shuppan; 1997 (in Japanese).

[18] Okuda N, Miura K, Yoshita K, Matsumura Y, Okayama A, Nakamura Y, Integration of data from NIPPON DATA80/90 and National Nutrition Survey in Japan: for cohort studies of representative Japanese on nutrition. J Epidemiol. 2010;20 Suppl 3:S506–14 10.2188/jea.JE20090218 20351471 PMC3920387

[19] Elwood PC, Pickering JE, Hughes J, Fehily AM, Ness AR Milk drinking, ischaemic heart disease and ischaemic stroke II. Evidence from cohort studies. Eur J Clin Nutr. 2004;58:718–24 10.1038/sj.ejcn.1601869 15116074

[20] Aboa-Eboulé C, Brisson C, Maunsell E, Mâsse B, Bourbonnais R, Vézina M, Job strain and risk of acute recurrent coronary heart disease events. JAMA. 2007;298:1652–60 10.1001/jama.298.14.1652 17925517

[21] Ballantyne C, Arroll B, Shepherd J Lipids and CVD management: towards a global consensus. Eur Heart J. 2005;26:2224–31 10.1093/eurheartj/ehi373 15972289

[22] Kondo I, Funahashi K, Nakamura M, Ojima T, Yoshita K, Nakamura Y; NIPPON DATA80/90 Research Group Association between food group intake and serum total cholesterol in the Japanese population: NIPPON DATA80/90. J Epidemiol. 2010;20 Suppl 3:S576–81 10.2188/jea.JE20090227 20351480 PMC3920384

[23] Jorde R, Bønaa KH Calcium from dairy products, vitamin D intake, and Blood pressure: Tromsø study. Am J Clin Nutr. 2000;71:1530–5 10837295 10.1093/ajcn/71.6.1530

[24] Snijder MB, van der Heijden AA, van Dam RM, Stehouwer CD, Hiddink GJ, Nijpels G, Is higher dairy consumption associated with lower body weight and fewer metabolic disturbances? The Hoorn Study. Am J Clin Nutr. 2007;85:989–95 17413097 10.1093/ajcn/85.4.989

[25] van Mierlo LA, Arends LR, Streppel MT, Zeegers MP, Kok FJ, Grobbee DE, Blood pressure response to calcium supplementation: a meta-analysis of randomized controlled trials. J Hum Hypertens. 2006;20:571–80 10.1038/sj.jhh.1002038 16673011

[26] Kesteloot H, Tzoulaki I, Brown IJ, Chan Q, Wijeyesekera A, Ueshima H, Relation of urinary calcium and magnesium excretion to blood pressure: The International Study Of Macro- And Micro-nutrients And Blood Pressure and The International Cooperative Study On Salt, Other Factors, And Blood Pressure. Am J Epidemiol. 2011;174:44–51 10.1093/aje/kwr049 21624957 PMC3159430

[27] Xu JY, Qin LQ, Wang PY, Li W, Chang C. Effect of milk tripeptides on blood pressure: a meta-analysis of randomized controlled trials. Nutrition. 2008;24:933–40 10.1016/j.nut.2008.04.004 18562172

[28] Sauvaget C, Nagano J, Allen N, Grant EJ, Beral V Intake of animal products and stroke mortality in the Hiroshima/Nagasaki Life Span Study. Int J Epidemiol. 2003;32:536–43 10.1093/ije/dyg151 12913025

[29] Kinjo Y, Beral V, Akiba S, Key T, Mizuno S, Appleby P, Possible protective effect of milk, meat and fish for cerebrovascular disease mortality in Japan. J Epidemiol. 1999;9:268–74 10.2188/jea.9.268 10510585

[30] Jenum AK, Stensvold I, Thelle DS Differences in cardiovascular disease mortality and major risk factors between districts in Oslo. An ecological analysis. Int J Epidemiol. 2001;30 Suppl 1:S59–65 10.1093/ije/30.suppl_1.S59 11759854

