# Supplementary material for: Consumption of Dairy Products and Death From Cardiovascular Disease in the Japanese General Population: The NIPPON DATA80
Source: J Epidemiol. 2013 Jan 5;23(1):47–54. doi: 10.2188/jea.JE20120054 (PMC3700229; doi:10.2188/jea.JE20120054)
Supplement: Abstract in Japanese. [file je-23-047-s001.pdf]

日本人の一般集団における乳製品の摂取と循環器疾患死亡：NIPPON DATA80

近藤今子[1,2]、尾島俊之[2]、中村美詠子[2]、早坂信哉[2]、寶澤篤[3]、斎藤重幸[4]、大西浩文[4]、赤坂憲[4]、早川岳人[5]、村上義孝[6]、奥田奈賀子[7]、三浦克之[8]、岡山明[9]、上島弘嗣[8,10]、NIPPON DATA80 研究グループ

[1]浜松大学健康プロデュース学部、[2]浜松医科大学健康社会医学講座、[3]東北メディカルメガバンク機構予防医学・疫学部門、[4]札幌医科大学内科学第二講座、[5]福島県立医科大学衛生学・予防医学講座、[6]滋賀医科大学医療統計学部門、[7]国立健康・栄養研究所栄養疫学研究部、[8]滋賀医科大学公衆衛生学部門、[9]日本結核予防会第一健康相談所、[10]滋賀医科大学生活習慣病予防センター

【背景】近年、牛乳・乳製品の摂取と循環器疾患との負の関連が西洋諸国の研究で報告されている。本研究では、日本における牛乳・乳製品の摂取と循環器疾患死亡との関連について検討した。

【方法】1980 年に日本全体の 300 地区で実施した国民栄養調査の対象者のうち 30 歳以上の男女を 24 年間追跡した。牛乳・乳製品摂取量の 3 分位間の死亡リスクをコックスの比例ハザードモデルにより高摂取群を基準として算出した。また、摂取量 1 日 100 g 増加あたりのハザード比も算出した。

【結果】対象者 9,243 人の 24 年間の追跡期間における循環器疾患死亡は 893 人で、そのうち心疾患死亡が 174 人、脳血管疾患死亡が 417 人であった。女性において、低摂取群の循環器疾患死亡、心疾患死亡、脳血管疾患死亡のハザード比は年齢、BMI、喫煙習慣、飲酒習慣、糖尿病歴、降圧剤服薬、職業区分および総エネルギー摂取量を調整した場合それぞれ 1.27(95% 信頼区間:0.99-1.58; トレンド p=0.045)、1.67(0.99-2.80;p=0.02)、1.34(0.94-1.90;p=0.08)であった。牛乳・乳製品 1 日 100 g 摂取増加毎のハザード比は、女性において循環器疾患死亡 0.86(0.74-0.99)、心疾患死亡 0.73(0.52-1.03)、脳血管疾患死亡 0.81(0.65-1.01)で低下傾向にあった。男性では有意な関連は見られなかった。

【結論】牛乳・乳製品の摂取は日本において女性で循環器疾患死亡と負の関連があった。

キーワード：乳製品、循環器疾患、死亡率、血圧、心疾患
